# Surgical Management of the Horizontal Instability of the Acromioclavicular Joint of a Young Athlete: A Case Report

**DOI:** 10.7759/cureus.59823

**Published:** 2024-05-07

**Authors:** John Grossi, George Ackerman

**Affiliations:** 1 Research, Lake Erie College of Osteopathic Medicine, Bradenton, USA; 2 Orthopedic Surgery, Optum Orthopedics and Sports Medicine, Lake Success, USA

**Keywords:** orthopedic surgery shoulder and elbow and upper extremity, horizontal instability, orthopedic sports medicine, acromioclavicular joint injuires, acromioclavicular joint

## Abstract

The acromioclavicular joint (ACJ) is commonly injured due to a force to the lateral aspect of the shoulder with the arm in adduction resulting in vertical or multidirectional instability. However, in this case report, we present a rare case of an athlete with isolated horizontal ACJ instability, which was treated surgically using a hamstring allograft. We highlight the surgical technique use to stabilize the joint in the horizontal (anterior to posterior) plane. Isolated horizontal instability of the ACJ is very rare and, to our knowledge, this surgical technique has not been reported in the case of a young athlete.

## Introduction

The acromioclavicular joint (ACJ) is a diarthrodial joint that is supported by the acromioclavicular (AC) and coracoclavicular (CC) ligaments [1]. The ACJ is a commonly injured upper extremity joint in young athletes [2]. According to Pallis et al., the incidence of ACJ injuries in a young athletic cohort was 9.2 injuries per 1000 persons-years [3]. The ACJ is supported by the CC ligament complex and the AC ligament complex [3]. The CC ligament complex is composed of the trapezoid and conoid ligaments, which make up the posteromedial and anterolateral portion of the complex, respectively [3]. ACJ injuries are commonly caused in a vertical plane and they are classified using the Rockwood Classification scale [4]. Type I and II injuries are generally treated conservatively, types IV and V injuries are often treated surgically, and type III injury treatments are based on the patient’s activity level, functional impairment, and aesthetic preferences [4].

Vertical stability of the ACJ is provided by the CC ligaments, while horizontal stability is provided by the AC ligament complex [5]. There are two common mechanisms of injury, direct and indirect [5]. Direct injuries are more common and constitute injuries where there is a direct fall or impact to the ACJ, while in indirect, there is a fall onto an outstretched hand, which leads to the humerus providing a superior force into the acromion [5]. These mechanisms of injury commonly lead to vertical instability of the ACJ rather than horizontal instability due to the AC ligament being stronger than the CC ligament, more specifically, the conoid ligament being the first to fail [5]. However, in this unique case, the patient was found to have isolated horizontal instability of the ACJ. Interestingly, his coracoclavicular ligaments remained intact, and he was noted to have attrition of the AC ligament complex, which is not the typical presentation given the usual mechanism of injury. 

## Case presentation

**Figure 1:**
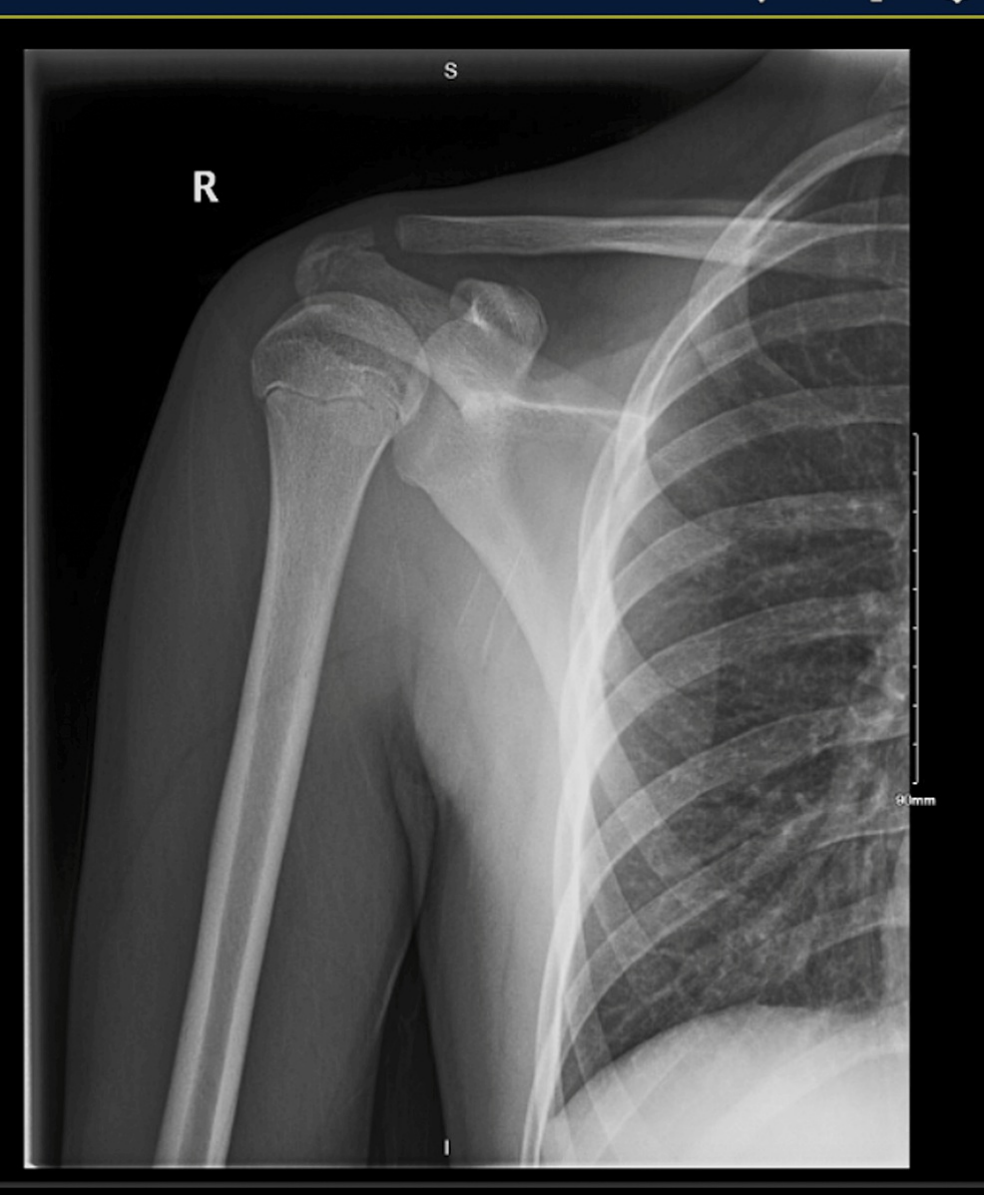
AP view of the right shoulder, no superior or inferior instability of the acromioclavicular joint

**Figure 2:**
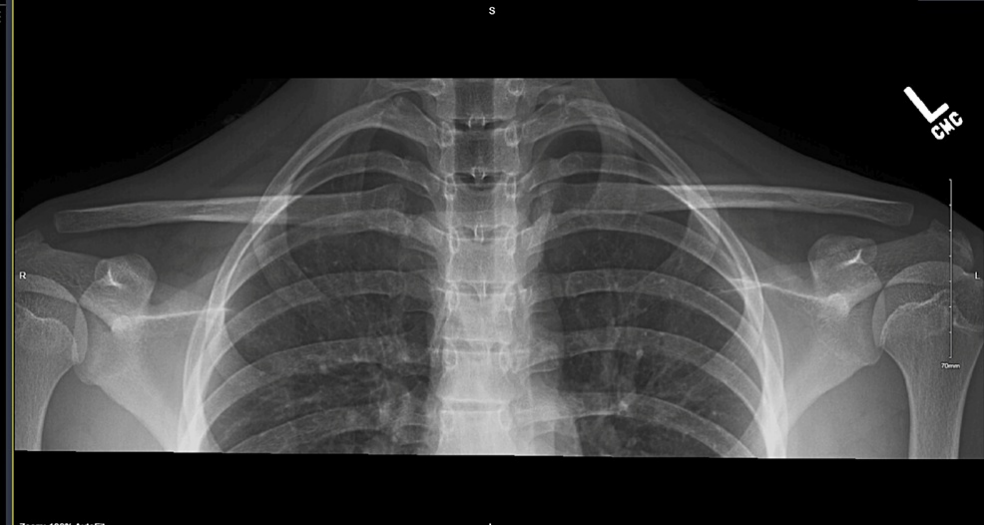
Bilateral view of the acromioclavicular joints, no superior or inferior instability of the acromioclavicular joints

A 15-year-old right-hand dominant male presented with right shoulder clicking and instability for approximately two months when an opponent fell onto his shoulder while playing football. He complained of pain radiating to his axilla as well as difficulty with overhead activities including throwing. X-rays of the shoulder in the AP and bilateral ACJ views, as shown in Figures 1, 2, were unremarkable. No surgical intervention was necessary at this time. After three months of conservative treatment with physical therapy to improve his range of motion, strengthening, and scapular mechanics, his horizontal AC instability did not improve. He was able to eliminate his symptoms only when his physical therapist stabilized his clavicle while throwing. His instability was noted to be both visible and palpable with overhead and throwing motions. Stress views of the ACJ were then taken, which revealed no superior or inferior instability of the ACJ. When re-reviewing his previous MRI with his radiologist, he was found to have atrophic changes and thinning of his ACJ capsule, which correlated with his horizontal instability. After several months of physical therapy, surgical intervention was deemed necessary to restore his horizontal ACJ stability. 

In surgery, the goal was to perform an AC ligament reconstruction with a gracilis allograft. A transverse incision was made overlying the ACJ. The clavicle was found to be very unstable in the horizontal plane with significant anterior and posterior motion at the ACJ. After the ACJ capsule was incised and dissected, a V-shaped 4 mm transosseous tunnel was created 1 cm lateral to the ACJ on the acromion. An anterior-to-posterior transosseous tunnel was then created 1.5 cm medial to the ACJ line in the clavicle. The gracilis allograft was then passed through the V-shaped tunnel on the acromion and then superiorly over the AC joint, creating a figure-of-eight configuration. The anterior limb of the graft was then passed through the clavicle from anterior to posterior and the posterior limb of the graft was then passed through the clavicle posterior to anterior. While maintaining tension on the graft as well as holding tension on the clavicle in a reduced position, a 3.9 mm SwiveLock C anchor (Arthrex, Inc., Naples, Florida, United States) was inserted through the clavicular tunnel from anterior to posterior securing the graft. Excellent fixation was noted. The graft was then brought back and tied over itself posteriorly superiorly for further fixation with FiberWire suture (Arthrex, Inc., Naples, Florida, United States). The ACJ was then found to be stable under direct visualization with manually directed stress, as shown in Video 1. 

**Video 1:** Preoperative and intraoperative acromioclavicular (AC) joint manipulation

## Discussion

Most published surgical technique case reports regarding ACJ reconstruction are focused on addressing vertical and multiplanar instabilities [6]. Before diving into the surgical technique of ACJ reconstruction for horizontal instability, confirming the diagnosis is the first step. It is important to consider both the vertical and horizontal planes when assessing the ACJ on a physical exam. Imaging studies are often helpful in confirming the clinical diagnosis of horizontal plane ACJ instability. The best radiographic view to evaluate the horizontal instability of the ACJ is a functional or stress axillary view [7]. Although not used in this particular case study, this view can be very helpful in assessing the horizontal complement of the instability pattern. 

There is no gold standard surgical procedure for isolated horizontal instability of the ACJ [8]. Given the lack of vertical instability, there is no reason to reconstruct the CC ligaments in cases of isolated horizontal ACJ instability. The goal of this surgical technique was to restore and reconstruct the AC ligament complex. The figure-of-eight configuration with a V-shaped acromial tunnel was preferred in this case. Despite low risks reported in the literature, a V-shaped acromial tunnel was used in an effort to avoid passing the graft through the subacromial space and potentially causing shoulder impingement [9]. This technique should be considered for overhead athletes to avoid possible impingement symptoms in the future. 

When assessing the ability of an athlete to return to sport status post ACJ reconstruction, important considerations are made when assessing the type of instability and the type of sport to which the athlete will return [10]. The mean time for return to play following an AC joint reconstruction was shown to be 5.7 months, however this was noted in vertical instability rather than horizontal [11]. In the same study, it has been shown that one fifth of the overhead athletes did not return to their preinjury levels [11]. In this case report, the patient was able to return to throwing and baseball activities at five months. 

## Conclusions

Horizontal instability of the ACJ is uncommon due to the superior strength of the AC ligament complex in comparison to the CC ligament complex. The presented case provides a unique technique to stabilize the ACJ in the horizontal plane. There is a paucity of literature on the surgical treatment of isolated ACJ horizontal instability; therefore, there is no gold standard procedure. The surgical technique presented in this case offers a novel and reliable method to restore the horizontal stability of the ACJ in the throwing athlete. 

## References

[1] Aliberti GM, Kraeutler MJ, Trojan JD, Mulcahey MK (2020). Horizontal instability of the acromioclavicular joint: a systematic review. Am J Sports Med.

[2] Mazzocca AD, Arciero RA, Bicos J (2007). Evaluation and treatment of acromioclavicular joint injuries. Am J Sports Med.

[3] Pallis M, Cameron KL, Svoboda SJ, Owens BD (2012). Epidemiology of acromioclavicular joint injury in young athletes. Am J Sports Med.

[4] Gorbaty JD, Hsu JE, Gee AO (2017). Classifications in brief: Rockwood classification of acromioclavicular joint separations. Clin Orthop Relat Res.

[5] Saccomanno MF, DE Ieso C, Milano G (2014). Acromioclavicular joint instability: anatomy, biomechanics and evaluation. Joints.

[6] Afonso J, Agneskirchner J (2021). Monoplanar horizontal instability of the acromioclavicular joint: case report and stabilization surgical technique. JSES Int.

[7] Tauber M, Koller H, Hitzl W, Resch H (2010). Dynamic radiologic evaluation of horizontal instability in acute acromioclavicular joint dislocations. Am J Sports Med.

[8] Aliberti GM, Mulcahey MK, Brown SM, O'Brien MJ (2020). Restoring horizontal stability of the acromioclavicular joint: open acromioclavicular ligament reconstruction and repair with semitendinosus allograft. Arthrosc Tech.

[9] Tauber M, Valler D, Lichtenberg S, Magosch P, Moroder P, Habermeyer P (2016). Arthroscopic stabilization of chronic acromioclavicular joint dislocations: triple- versus single-bundle reconstruction. Am J Sports Med.

[10] Kay J, Memon M, Alolabi B (2018). Return to sport and clinical outcomes after surgical management of acromioclavicular joint dislocation: a systematic review. Arthroscopy.

[11] Cleary BP, Hurley ET, Kilkenny CJ (2024). Return to play after surgical treatment for acromioclavicular joint dislocation: a systematic review. Am J Sports Med.

